# Chronic Condition Discordance and Physical Activity: Longitudinal Associations Among Older Couples

**DOI:** 10.1093/geroni/igaa057.2044

**Published:** 2020-12-16

**Authors:** Courtney Polenick, Kira Birditt, Angela Turkelson, Helen Kales

**Affiliations:** 1 University of Michigan, Ann Arbor, Michigan, United States; 2 UC Davis, Sacramento, California, United States

## Abstract

Chronic condition discordance (i.e., the extent that two or more conditions have non-overlapping self-management requirements) is detrimental for functional health but little is known about mechanisms accounting for these associations. We examined links between chronic condition discordance at both the individual level and the couple level (i.e., between spouses) and physical activity over time. Participants included 1,095 couples from five waves (2006-2014) of the Health and Retirement Study. Dyadic growth curve models showed that greater individual-level discordance was associated with lower baseline physical activity among individuals and their partners. When husbands had greater individual-level discordance, wives and husbands had faster declines in physical activity. The findings highlight the importance of considering both members of a couple when examining the implications of chronic illness for physical activity in middle and later life.

